# Phosphorylation of Initiation Factor eIF2 in Response to Stress Conditions Is Mediated by Acidic Ribosomal P1/P2 Proteins in *Saccharomyces cerevisiae*

**DOI:** 10.1371/journal.pone.0084219

**Published:** 2013-12-31

**Authors:** Antonio Jiménez-Díaz, Miguel Remacha, Juan P. G. Ballesta, Juan José Berlanga

**Affiliations:** Centro de Biología Molecular Severo Ochoa, Consejo Superior de Investigaciones Científicas and Universidad Autónoma de Madrid (CSIC-UAM), Madrid, Spain; The John Curtin School of Medical Research, Australia

## Abstract

Eukaryotic cells contain an unusually large cytoplasmic pool of P1/P2 phosphoproteins, which form the highly flexible 60S subunit stalk that is required to interact with and activate soluble translation factors. In cells, cytoplasmic P1/P2 proteins are exchanged for ribosome-bound proteins in a process that can modulate ribosome function and translation. Here, we analysed different *S. cerevisiae* stalk mutants grown under stress conditions that result in eIF2α phosphorylation. These mutants either lack a cytoplasmic pool of stalk proteins or contain free but not ribosome-bound proteins. Only cells that contain free P1/P2 proteins induce eIF2 phosphorylation in vivo in response to glucose starvation or osmotic stress. Moreover, we show that free *S. cerevisiae* P1/P2 proteins can induce in vitro phosphorylation of the initiation factor eIF2 by stimulating the autophosphorylation and activation of GCN2 kinase. Indeed, these ribosomal proteins do not stimulate other eIF2α kinases, such as PKR and HRI. P1/P2 and the known GCN2 activator deacylated tRNA compete for stimulating the eIF2α kinase activity of GCN2, although the P1/P2 proteins are considerably more active. These findings reveal a capacity of free cytoplasmic ribosomal stalk components to stimulate eIF2α phosphorylation, which in turn would modulate translation in response to specific forms of stress that may be linked with the previously described regulatory function of the ribosomal stalk.

## Introduction

The ribosomal stalk is a lateral protuberance of the large ribosomal subunit, which is essential for ribosome function in organisms of all biological Kingdoms. The stalk is formed by a set of 12 kDa acidic protein dimers that bind to a larger core protein, which in turn interacts with the highly conserved GTPase-associated region (GAR) of the large rRNA that binds the entire complex to the ribosome (see [1] for a review). In eukaryotes, the 32 kDa core protein P0 binds to two heterodimers of the 12 kDa P1 and P2 proteins, ultimately forming the stalk P0-(P1/P2)_2_ complex [2]. Some eukaryotic species possess more than one form of P1 and P2 proteins [3], [4], and a third family of acidic P proteins, P3, has been described in plants [5]. *S. cerevisiae* contains two isoforms of each protein [6], [7], [8], [9], currently termed P1α, P1β, P2α and P2β [10]. In contrast to bacteria, the acidic 12 kDa proteins of the yeast eukaryotic stalk are not essential for stalk function but rather, they modulate ribosomal activity [11].

There is solid evidence showing that P proteins perform their cellular functions as part of the stalk [12], [13], [14], [15]. However, several findings suggest additional roles for free P1/P2 proteins in the cell. Unbound acidic proteins were recently shown to affect the sensitivity of the yeast ribosome to certain ribosome inactivating proteins (RIPs) [16]. Indeed, an important feature of eukaryotic cells is their large cytoplasmic pool of free P1 and P2 proteins, which are exchanged with ribosomal-bound proteins in a process that is protein synthesis-dependent [2]. This exchange implies that at a yet undefined stage of translation, a stalk assembly/disassembly cycle may exist that facilitates the generation of ribosomes with defective stalk compositions [17], [18], these having a central role in proposed translation regulatory mechanisms [2].

There is experimental evidence that stalk composition affects different cellular processes. Yeast strains deprived of P1 and P2 stalk proteins are unable to sporulate [11], and P1/P2 depletion favours the internal initiation of translation in human cell lines [19]. Moreover, mitochondrial stability in yeast is dependent on the presence of these proteins (Camargo and Remacha, unpublished data). It is very likely that these alterations are due to stalk-dependent changes in the expression of proteins involved in these cellular pathways. To determine the mechanism by which the stalk affects the expression of specific proteins, we investigated the function of this ribosomal domain on the activity of translation factors.

Since it was initially reported, the direct involvement of the stalk in the activity of the bacterial elongation factor EFG has been thoroughly studied [20]. Moreover, although it is not completely understood there has been important progress made in determining the high resolution structure of the bacterial stalk [21]. The involvement of the bacterial stalk in the activity of initiation factor IF2 has also been described [22], [23], although significant advances in this field have only been made recently [24], [25]. Our current understanding of the role of the stalk in functional interactions with translation factors in eukaryotes is considerably poorer than that of bacteria. While the involvement of the stalk in the function of elongation factor EF2 has been reported [26], experimental data regarding its association with initiation factors are lacking.

Initiation factors, and particularly eukaryotic initiation factor 2 (eIF2), play well-documented roles in a number of eukaryotic mechanisms of translational regulation [25]. In all eukaryotes, and especially in *S. cerevisiae*, specific phosphorylation of the α-subunit of translation initiation factor 2 (eIF2α) by eIF2α kinases is an important event in the regulation of protein synthesis in response to a variety of environmental stresses. This modification leads to a general inhibition of translation while enhancing the translation of specific messenger RNAs that, encoding transcription factors, stimulate the expression of genes involved in the cellular response to stress, such as *GCN4* in yeast and *ATF4* in mammals, thereby promoting cell survival in conditions of stress [27].

Gcn2, the only eIF2α kinase present in *S. cerevisiae*, is activated by amino acid or glucose starvation, or by other stressors such as osmotic stress [27]. Gcn2 is a highly conserved protein that is the sole eIF2α kinase expressed in most eukaryotic organisms and, in mammals, it has been implicated in essential functions such as the antiviral response and memory formation [28], [29]. In addition to the kinase region, Gcn2 contains distinct functional domains that regulate its activity and cellular localization. A domain adjacent to the kinase region resembles the histidyl-tRNA synthetases (HisRS) and binds uncharged tRNAs that accumulate upon amino acid deprivation, leading to the activation of the catalytic domain. At the C terminus, Gcn2 contains a region required for ribosome association and dimerization [30], [31].

Based on this information, we investigated the effect of stalk alterations on eIF2activity and on cellular responses to stress, and we found that free P1 and P2 proteins directly affect the phosphorylation of eIF2α by GCN2 kinase.

## Results

### Stress Response of *S. cerevisiae* Mutants with Altered Ribosomal Stalk Composition

**Figure 1:**
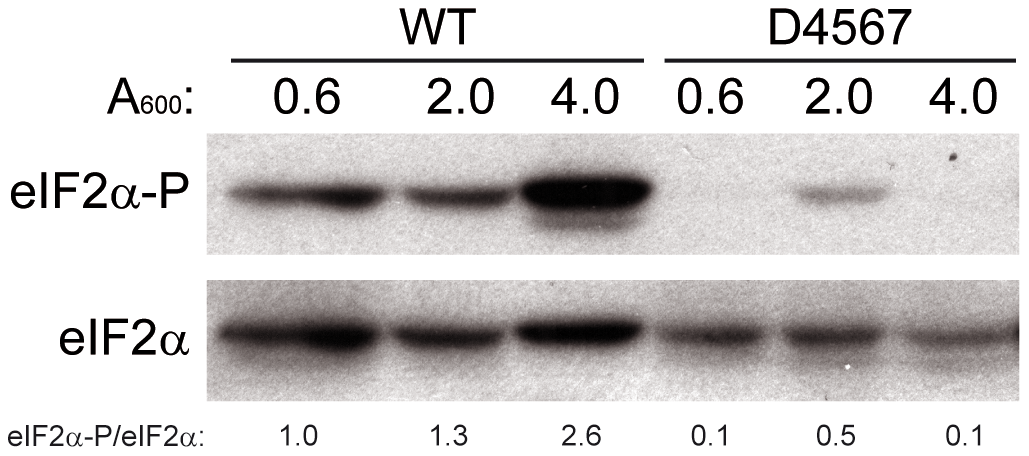
eIF2α phosphorylation at stationary phase in wild-type and in a mutant strain lacking P1/P2 proteins. *S. cerevisiae* W303-1b (WT) and mutant strain D4567 were grown up to stationary phase (A_600_ = 4.0) and the amount of phosphorylated and total eIF2α was estimated in total cell extracts resolved by SDS-PAGE. Phosphorylated and total eIF2α (eIF2α-P and eIF2α) were analyzed in Western blots probed with specific antibodies. Similar results were obtained from duplicate experiments. The values under Western blot panels represent the intensities of phosphorylated eIF2α in each lane normalized respect to the corresponding total eIF2α; for comparison, the value obtained in the first lane (WT A_600_ = 0.6) was set as 1. Similar results were obtained from duplicate experiments.

We have analyzed the response of *S. cerevisiae* stalk mutants with defective ribosomal stalks to a variety of growth conditions. We initially tested the extent of eIF2α modification along the growth curve in the D4567 strain, which totally lacks acidic proteins [11], and in the parental wild-type (W303-1b) strain. Dramatic differences were observed in both the exponential and the stationary phases of growth: while the level of eIF2α phosphorylation was markedly increased in wild-type cells during the stationary phase, it was very low in the exponential phase and only a modest increase was observed in D4567 cells in the A_600_ = 2.0 point (Figure 1).

These results led us to investigate the response of the yeast stalk mutants to stress conditions, which are known to affect initiation factor modification [32], [33], [34]. In these experiments, we included two additional strains, D67 and D45 that lack the P1 and P2 genes, respectively [35]. The ribosomes of these strains lack the acidic proteins, as acidic proteins must bind as P1/P2 heterodimers [35], [36] and only one of the partners is present in the cell. However, as P1 but not P2 proteins are quickly degraded in the absence of their respective partners [37], strain D67 harbours a cytoplasmic pool of unbound P2, whereas D45 contains no free acidic proteins. Conversely, like all eukaryotic cells [2], the wild-type strain contains a complete ribosomal stalk and a significant cytoplasmic pool of the four P1 and P2 protein isoforms.

The four yeast strains were grown under three commonly used conditions of stress: in the presence of 0.5 M NaCl, in the absence of glucose, and in conditions of amino acids deprivation. Previously, we confirmed that wild-type cells responded to these conditions by increasing eIF2α phosphorylation [32], [33], and that this effect is dependent on the presence of Gcn2 kinase (Figure S1).

**Figure 2:**
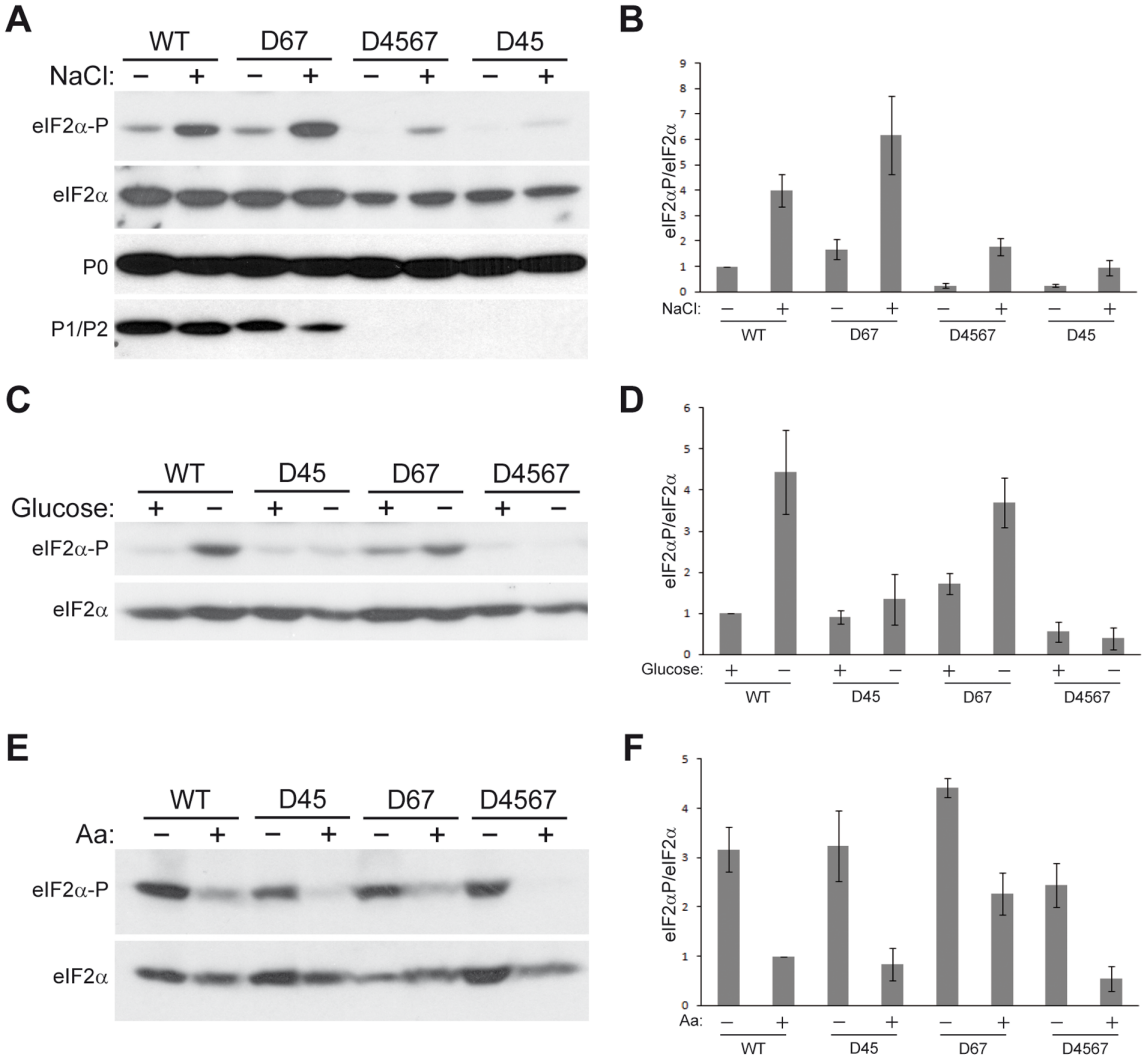
Response of *S. cerevisiae* stalk mutants to different stresses. Yeast D45, D67 and D4567 and the parental W303-1b (WT) strains were grown in the presence (+) or absence (–) of 0.5 M NaCl (A); in the presence of 2% (+) or 0.5% (–) glucose (C); or in the presence (+) or absence (–) of amino acids (E), as described in the Materials and methods section. After the appropriate period, the cells were collected, the total cell extracts were resolved by SDS-PAGE and the amount of phosphorylated and total eIF2α, ribosomal acidic proteins P0 and P1/P2, was analyzed as described in Fig. 1. (B, D, F) Quantification of the levels of phosphorylated eIF2α in response to stress. Values represent the ratio eIF2α-P/eIF2α in each case, referred to the values obtained in WT unstressed cells, which were set as 1. The results show the means of two independent experiments plus the standard deviations.

Afterwards, the amount of phosphorylated and total eIF2α was estimated in cell extracts from the four yeast strains in each of the three stress conditions (Figure 2). The expected increase in the eIF2α-P/eIF2α ratio was observed in extracts from the wild-type strain in the first two conditions, however this effect was notably attenuated (osmotic stress, Figure 2 A, B) or not detected at all (glucose deprivation, Figure 2 C, D) in D45 and D4567 extracts. By contrast, increased eIF2α phosphorylation was observed in the D67 strain, whose response resembled that seen for wild-type strain (Figure 2 A, B, C, D). All four strains responded comparably to amino acid starvation provoked by both absence of all the amino acids in the growth medium (Figure 2 E, F) or by 3-amino-1,2,4-triazole (3-AT) treatment (Figure S2).

**Figure 3:**
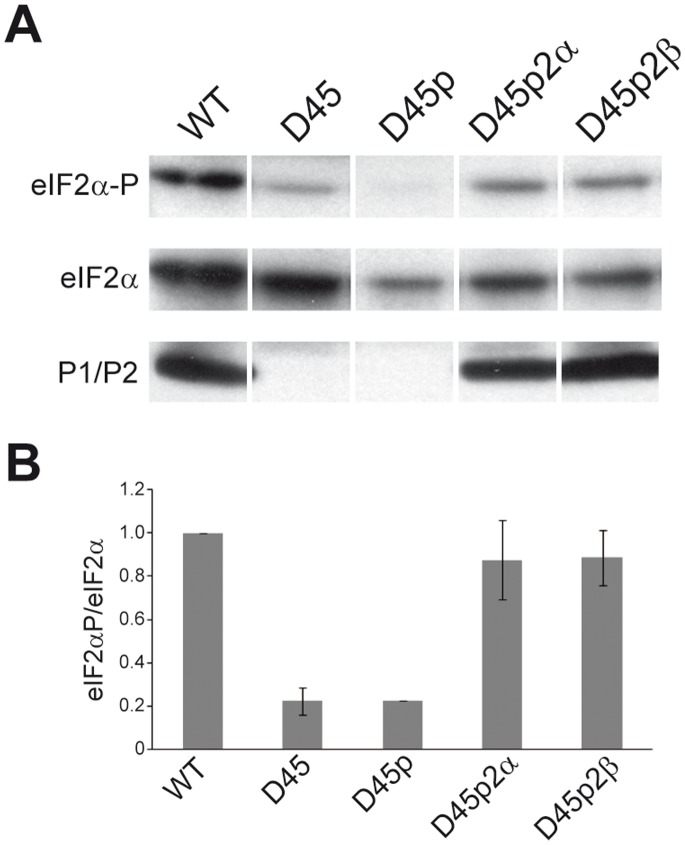
Ectopic expression of ribosomal stalk acidic proteins restores basal levels of eIF2α phosphorylation. (A) Cells of yeast strains W303-1b (WT), D45, D45p (transformed with pFL36), D45p2α (transformed with pFL36 P2α) and D45p2β (transformed with pFL36 P2β) growing in the mid-exponential phase (A_600_ = 0.6) were harvested and the levels of eIF2α-P, eIF2α and ribosomal acidic proteins P1/P2 in the cell extracts were analyzed as described in Fig. 1 (B) Quantification of the basal levels of phosphorylated eIF2α in each strain. Values represent the ratio eIF2α-P/eIF2α in each case, referred to the values obtained in WT cells, which were set as 1. The results show the means of two independent experiments plus the standard deviations.

Given that the presence of free stalk proteins seems to be associated to normal eIF2α phosphorylation levels (D67 strain), we studied the effect of generating a cytoplasmic pool of these proteins in a strain which totally lacks them (D45 strain). We expressed either P2α or P2β in D45 cells to induce the presence of ribosome bound and free P1/P2 dimers and the basal phosphorylation state of eIF2α was analysed (Figure 3). As can be observed, the expression of either P2 protein was sufficient to restore eIF2α phosphorylation to the same level of wild-type cells.

### Phosphorylation of eIF2α by GCN2 is Strongly Stimulated in Vitro by Ribosomal Stalk Proteins P1 and P2

**Figure 4:**
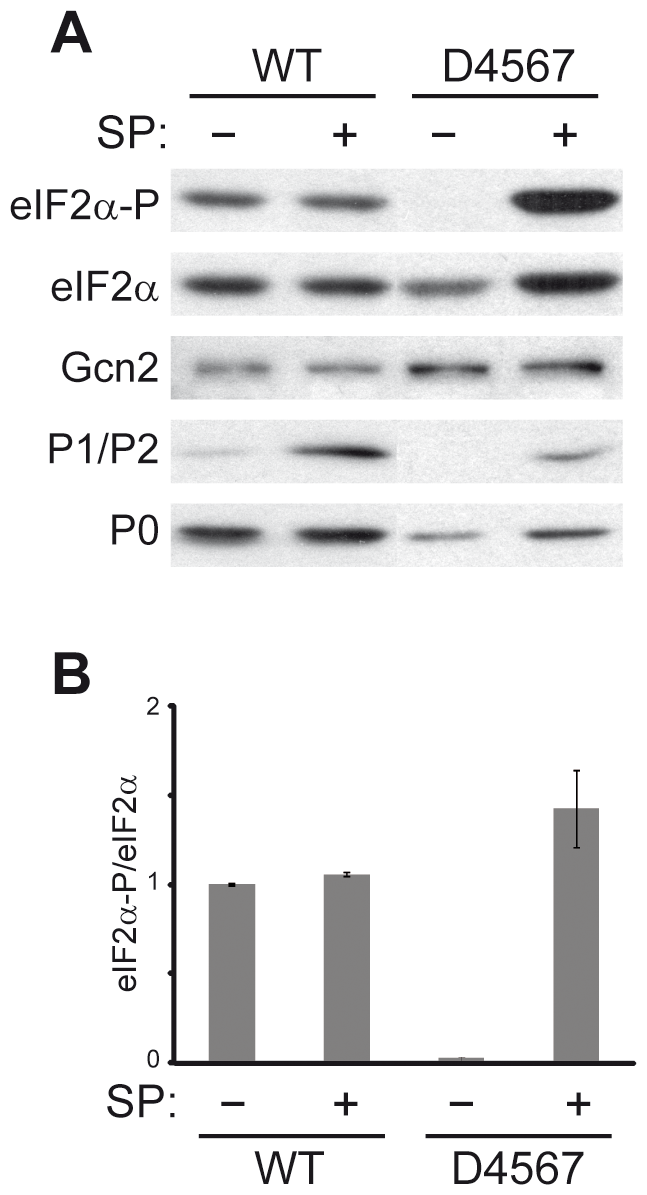
Ribosomal stalk acidic P1/P2 proteins stimulate the phosphorylation of eIF2α in a yeast cell-free *in vitro* translation system. Yeast cell-free translation extracts from W303-1b strain (WT) and D4567 were assayed as described in Materials and methods, in the presence (+) or absence (–) of purified P1/P2 proteins (SP). (A) Equivalent aliquots of all the assays were analysed by Western blot in order to detect phosphorylated (eIF2α-P) and total eIF2α, GCN2, and ribosomal acidic proteins (P0, P1/P2). (B) Quantification of the levels of phosphorylated eIF2α in response to the presence of P1/P2 acidic proteins. Values represent the ratio eIF2α-P/eIF2α in each case, referred to the values obtained in WT extract without added P1/P2 proteins, which were set as 1. The results show the means of two independent experiments plus the standard deviations.

The response of the different yeast mutants to osmotic stress and glucose deprivation pointed to a possible role of the free cytoplasmic stalk proteins on the eIF2α phosphorylation mediated by the eIF2α kinase Gcn2. This possibility was directly tested by adding the split protein fraction (SP) obtained after washing ribosomes with 0.3 M NH_4_Cl/50% ethanol [26], which essentially contains only P1/P2 proteins [9] to an in vitro cell-free translation extract (Figure 4). A significant increase in eIF2α phosphorylation was observed upon addition of SP to P1/P2-deprived cell extracts from strain D4567, but not to preparations from wild-type cells, which displayed in contrast higher basal levels of phosphorylated eIF2α. These results suggest that the presence of P1/P2 proteins promote the phosphorylation of eIF2α by Gcn2, the only eIF2α kinase present in yeast cells.

**Figure 5:**
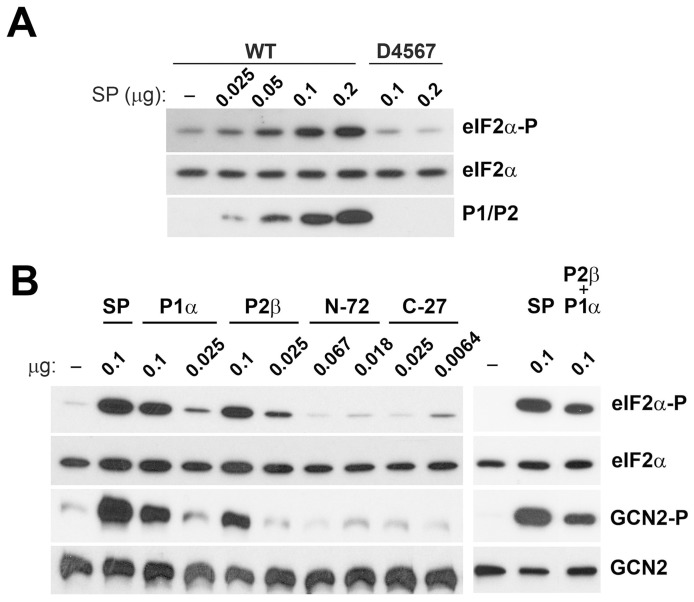
Ribosomal stalk proteins stimulate the phosphorylation of eIF2α by GCN2 kinase *in vitro*. (A) Increasing amounts of a P1/P2 ribosomal extract (SP fraction) from either wild-type W303-1b (WT) or mutant D4567 were added to a phosphorylation assay containing purified eIF2α and GCN2 kinase. (B) The indicated amounts of P1α and P2β recombinant proteins, and equimolecular amounts of the P2β NTD and CTD polypeptides, were added to a GCN2-dependent eIF2α phosphorylation assay. In a parallel assay, a mixed equimolecular total amount of both proteins (0.05 µg of each protein, P1α and P2β) were also tested. The same amount of SP extract (0.1 µg) was used as a control. In both cases, following kinase assay, the samples were resolved by SDS-PAGE, and the amount of phosphorylated (eIF2α-P) and total eIF2α, phosphorylated GCN2 (GCN2-P) and total GCN2, and ribosomal acidic proteins (P1/P2) were estimated by Western blot. Similar results were obtained from duplicate experiments.

To corroborate these results we added increasing amounts of the split protein fraction (SP), obtained from either wild-type or P1/P2 proteins-depleted ribosomes, to an in vitro assay containing only purified eIF2 and mouse GCN2, which has been shown structurally [38] and functionally [28] equivalent to yeast Gcn2. Strong stimulation of eIF2α phosphorylation by GCN2 was observed in the presence of the acidic proteins (Figure 5A), whereas a similar fraction from ribosomes lacking P1/P2 proteins produced no such stimulation. Indeed, the stimulatory effect of the stalk proteins was confirmed by adding to the kinase assay recombinant P1α or P2β proteins purified by standard chromatography [39]. The addition of these proteins strongly stimulated eIF2α phosphorylation, although slightly less than the SP fraction containing all the P1/P2 proteins (Figure 5B).

To precisely identify the domain involved in GCN2 activation and subsequent eIF2α phosphorylation, the effects of the fragments comprising the first 72 amino acids (N-72) or the last 27 amino acids (C-27) of the protein P2β were analyzed in the in vitro initiation factor phosphorylation assay (Figure 5B). Quantities of fragment equimolecular to that of the full protein were used, of which only the fragment of the Carboxy-Terminal Domain (CTD) produced a very mild stimulatory effect on eIF2α phosphorylation. Moreover, this already weak effect was even reduced when the amount of fragment added was increased 4-fold, perhaps due to the aggregation of peptides or to negative side effects at higher concentrations. Unfortunately, these results do not allow us to draw definitive conclusions and further work will be required to determine the precise P1/P2 protein region responsible for GCN2 activation.

Acidic stalk proteins are usually present in the cell and form P1/P2 heterodimers. However, eIF2α phosphorylation was not significantly increased when both purified proteins were added together, suggesting that the formation of P1/P2 heterodimers is not essential for their stimulatory effect (Figure 5B).

As autophosphorylation seems to be important for GCN2 activity [40], the effect of the ribosomal proteins on the protein kinase was also assessed using a specific antibody against the modified threonine-898 residue [41], which revealed acidic protein-dependent stimulation of GCN2 autophosphorylation closely correlated to the eIF2α phosporylation stimulation (Figure 5B).

### P1/P2 Compete with tRNA to Stimulate eIF2α Phosphorylation

**Figure 6:**
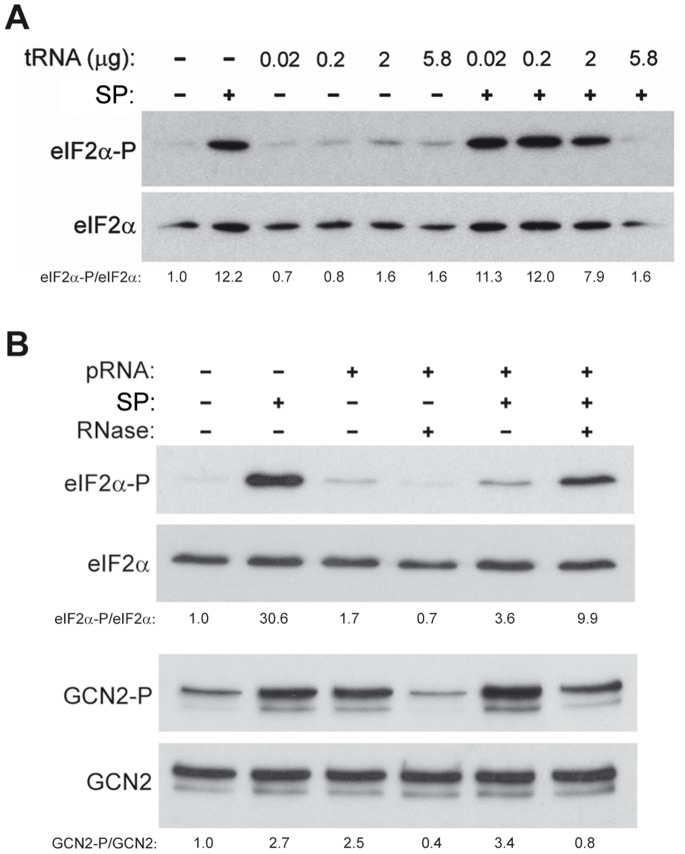
Effect of RNA on stimulation of eIF2α phosphorylation by P1/P2 proteins. (A) Stimulation of eIF2α phosphorylation by P1/P2 proteins is blocked by tRNA. The effect of increasing amounts of tRNA (0.02 µg ≈ 1 pmol, 0.2 µg ≈ 10 pmol, 2 µg ≈ 100 pmol, 5.8 µg ≈ 230 pmol) on GCN2-dependent eIF2α phosphorylation was tested in a kinase assay in the presence or absence of the SP fraction (0.1 µg ≈ 10 pmol), as described in Fig. 5. (B) RNase abolishes the inhibitory effect of RNA on P1/P2-mediated eIF2α phosphorylation. Polysomal RNA (pRNA, 11 µg ≈ 11 pmol), P1/P2 proteins (SP fraction, 0.1 µg) and a RNase mix (2 µg/ml RNase A, 0.1 µg/ml RNase T1, 12.5 µg/ml Micrococcal Nuclease S7, 0.125 mM CaCl_2_) were added to the *in vitro* GCN2-dependent eIF2α phosphorylation assay as indicated, and the samples were processed as described in A. The level of GCN2 autophosphorylation was also estimated using specific antibodies. The values under Western blot panels represent the intensities of phosphorylated proteins in each line normalized respect to the corresponding total proteins; for comparison, the value obtained in the first line (negative controls) was set as 1. Shown are the results of a representative experiment of other with similar results.

Phosphorylation of eIF2α by Gcn2 kinase is stimulated by RNA and more specifically, by uncharged tRNA [42]. The histidyl-tRNA synthetase-related sequence in GCN2 interacts with tRNA, which is required for Gcn2 activation in response to starvation of different amino acids [42]. To determine whether nucleic acid molecules and P1/P2 proteins stimulate eIF2α phosphorylation via the same mechanism, we examined the effect of adding increasing concentrations of tRNA to the eIF2α phosphorylation assay. Ribosomal proteins exhibited markedly higher stimulatory activity than tRNA, while the addition of tRNA attenuated the stimulatory effect of P1/P2 (Figure 6A). Similar results were obtained using yeast purified polysomal RNA (pRNA). While the nucleic acid molecules induced less eIF2α phosphorylation than the ribosomal proteins, when both elements were added in combination eIF2α phosphorylation was attenuated (Figure 6B). This reduction in eIF2α phosphorylation was due to the presence of RNA since P1/P2 stimulation was recovered when RNA was degraded by adding RNase to the reaction. As expected, RNase treatment totally abolished the stimulatory effect of RNA alone, but did not affect much P1/P2 action (Figure 6B).

Interestingly, the RNA and the P1/P2 proteins induced comparable increase in GCN2 autophosphorylation and appeared not to compete in this process. Moreover, an increase in kinase phosphorylation was detected when both effectors were added in combination, while the addition of RNase attenuated the activation due to degradation of the RNA molecules (Figure 6B).

### Effects of P1/P2 Stalk Proteins and tRNA on Different GCN2 Mutants

**Figure 7:**
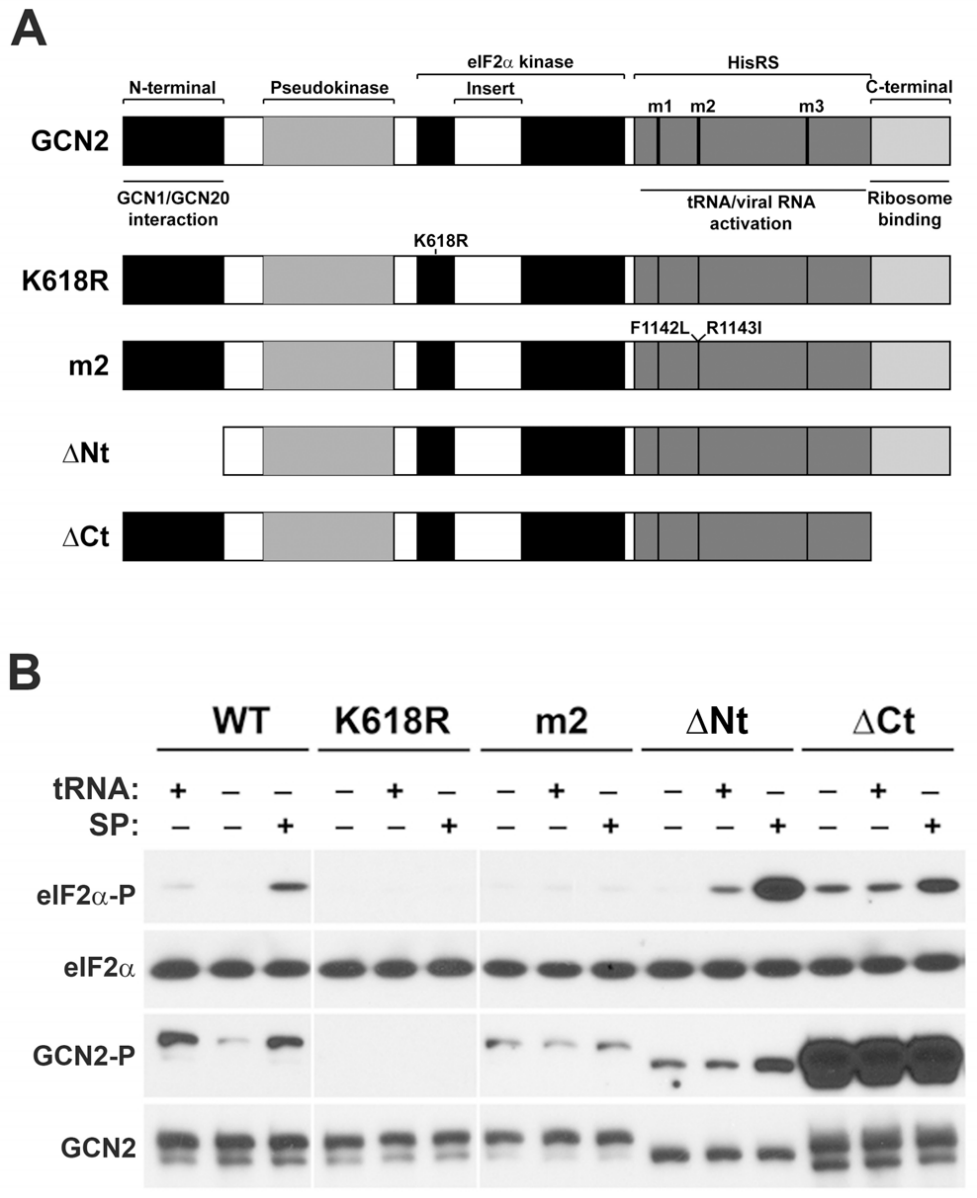
Effect of P1/P2 proteins and tRNA on the activity of the GCN2 mutants. (A) Schematic representation of the structural domains of GCN2 and mouse GCN2 mutants tested in this assay, indicating the punctual mutations or the deletions for each mutant. The full length GCN2 sequence is illustrated by a larger box. The figure is drawn to scale. Highlighted domains include the N-terminal (black box); the ‘Pseudokinase’ (grey box) that is related to subdomains I–XI of eukaryotic protein kinases; the conserved two lobes of the eIF2α kinase domain (black), separated by a large insert (white box); the HisRS-like domain (dark grey box) that includes the three motifs (m1, m2 and m3) conserved among the class II aminoacyl-tRNA synthetases; and a C-terminal domain (clear grey box). (B) Phosphorylation of eIF2α by GCN2 and the indicated GCN2 mutants was tested in the presence or absence of either tRNA (2 µg) or the SP fraction (0.1 µg) as previously described. Similar results were obtained from duplicate experiments.

To further investigate the competition between stalk proteins and tRNA in stimulating GCN2 activity, we analyzed the effect of both effectors on different GCN2 mutants (Figure 7A) described in Materials and methods. When eIF2α phosphorylation was analysed, mutations at either the kinase active domain (K618R mutant) or the tRNA activation domain (m2 mutant) totally abolished the stimulatory effect of both tRNA and P1/P2 on GCN2 activity (Figure 7B). These results, along with those shown in Figure 6A, suggest that both acidic proteins and tRNA induce GCN2 activation via a mechanism involving the HisRS domain of the kinase. By contrast, when the GCN2 amino-terminus first 200 amino acids, which are involved in the interaction of GCN2 with the GCN1-GCN20 complex, were deleted (ΔNt mutant) a significant increase in GCN2-dependent eIF2α kinase activity was induced by both effectors. Conversely, deletion of the GCN2 carboxy-terminus last 156 amino acids (ΔCt mutant), required for binding to the ribosome, which increases background GCN2 autophosphorylation, did not alter the effect of P1/P2 and tRNA on its eIF2α kinase activity to a great extent.

### Acidic Stalk Proteins do not Stimulate eIF2α Phosphorylation by other eIF2α Kinases

**Figure 8:**
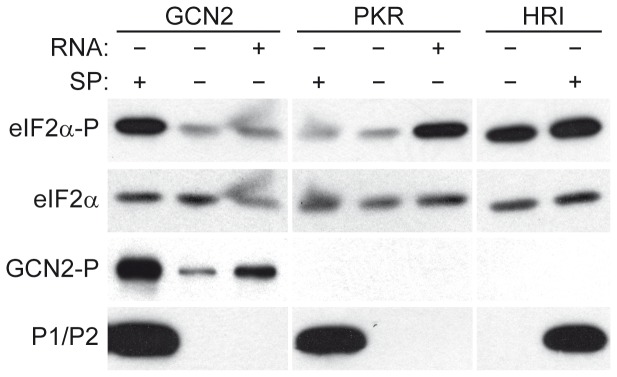
Effect of P1/P2 proteins on eIF2α phosphorylation by the kinases GCN2, PKR and HRI. Affinity-purified protein kinases were subjected to eIF2α kinase assay in the presence or absence of P1/P2 (SP fraction, 0.1 µg) and SV RNA (0.1 µg ≈ 0.03 pmol). The samples were analyzed after incubation by electrophoresis and Western blot as described in the previous figures. Similar results were obtained from duplicate experiments.

In addition to GCN2, three additional protein kinases that specifically phosphorylate eIF2α have been described in eukaryotic cells, namely PKR, PERK and HRI [43]. However, when the effect of the P1/P2 proteins on the capacity of PKR, HRI and GCN2 to modify eIF2 was assessed, eIF2α phosphorylation by either PKR or HRI was not significantly affected by the presence of acidic stalk proteins (Figure 8). It should be noted that the amount of viral RNA required to strongly stimulate PKR-dependent eIF2α phosphorylation has only a mild effect on GCN2. These results indicate that acidic stalk proteins specifically activate GCN2 but no other eIF2α kinases, and that the weak response of this kinase to the different RNA molecules tested, when compared to that produced by the P1/P2 proteins, is due to the greater stimulatory activity of the ribosomal proteins and not to the inactivation of the nucleic acid effector.

## Discussion

Extra-ribosomal functions of ribosomal components have been described previously [44], and a recent review questioned why these functions are observed less frequently than expected, considering the key role of the ribosomal proteins in controlling overall cell metabolism [45]. As the cytoplasmic pool of P1/P2 proteins is large and experimental evidence indicates that the free stalk proteins participate in the intracellular stimulation of some ribosome-inactivating proteins (RIP) [16], an additional extra-ribosomal function of these proteins may not be totally unexpected [46].

The high level of eIF2α phosphorylation observed at the stationary phase in wild-type cells in contrast to the P1/P2-deprived D4567 strain is compatible with a role in the modification of eIF2α for the P1/P2 ribosome stalk proteins, which accumulate in wild-type and are missing in the mutant strain. However, the response of *S. cerevisiae* stalk mutants to stress conditions is more compelling. The D67 strain, which contains ribosomes totally depleted of acidic proteins and that carries a pool of free P2 proteins, responds to glucose depletion and osmotic stress by increasing eIF2α phosphorylation, as also seen in the wild-type strain. By contrast, under the same growth conditions no changes in eIF2α phosphorylation were observed in the D45 and D4567 strains, both also carrying ribosomes depleted of acidic proteins but in addition lacking a free protein pool. The distinct responses of the three stalk mutants strongly suggest that free stalk proteins, present in D67 and absent in D45 and D4567, are directly involved in eIF2α phosphorylation in vivo. In fact, the expression of just one of the P2 missing proteins, which induced the generation of a cytoplasmic pool of P1/P2 heterodimers, is sufficient to raise the low basal eIF2α phosphorylation level of D45 strain at levels comparable to those of the wild-type strain.

Supporting this conclusion, we demonstrate here that the yeast acidic stalk proteins P1 and P2 can stimulate phosphorylation of the α-subunit of the eIF2 translation initiation factor in yeast cell-free translation extracts. Moreover, these proteins promote GCN2 autophosphorylation and induce eIF2α phosphorylation when tested in vitro using purified components. Additionally, the specificity of the stalk proteins for GCN2 activation, without affecting the activity of other well-known eIF2α kinases like PKR or HRI [47], supports the biological significance of the reported induction of eIF2 phosphorylation, as GCN2 is the only eIF2α kinase present in most eukaryotic organisms. Then, it is reasonable to conclude that this functional interaction could be a mechanism of translation regulation present not only in *S. cerevisiae*, but also in other eukaryotic cells. Remarkably, the ribosomal P1/P2 proteins are considerably more active than the tRNA, which is known to be a natural activator of GCN2 in vivo [42], although both effectors seem to stimulate the eIF2α kinase activity acting through the HisRS domain. Thus, the nucleic acid molecule inhibits ribosomal protein stimulation of GCN2 activity, whereas a number of GCN2 mutants carrying alterations in different regions of the protein respond similarly to the presence of both effectors.

The very mild induction observed when the CTD of the P2β protein was analyzed in the eIF2α phosphorylation assay could suggest that this highly conserved region may contribute to the stimulatory effect of P1/P2. Indeed, the highly mobile CTD is the functional part of the acidic stalk proteins [21]. Nonetheless, the complete molecule is required for optimal GCN2 stimulation an other parts of the protein are clearly required for full activity, either through direct action or by inducing the appropriate conformation in the CTD. The slightly higher activity of the SP preparations, which contained native proteins, as opposed to the recombinant purified proteins, also suggests that the correct protein conformation is required. This conformation may not be fully re-established after the denaturing process used to purify the recombinant proteins tested.

The stimulation of Gcn2 by deacylated–tRNA involves several reinforcing conformational transitions of the kinase domain (PK) initiated by disruption of the interaction between the active PK domain and the ribosome binding domain (RB/DD). Unfortunately, the information on the structure of the tRNA binding site on Gcn2 is scarce, apart from the fact that it might involve Glu803 [48], [49]. On the other hand, the available information on the structure of the P1/P2 proteins, mainly limited to their NTD [50], exclude an overall structural resemblance to the tRNA. Therefore, it is presently very difficult to propose a mechanism to explain the GCN2 stimulatory effect of the free ribosomal stalk proteins. An interesting observation related to the activation mechanism of the kinase, is that whereas RNA and P1/P2 proteins induce similar levels of GCN2 phosphorylation and could collaborate on that effect, they seem, however, to compete in the stimulation of eIF2α phosphorylation. This means that RNA and P1/P2 proteins could induce GCN2 phosphorylation acting through the same domain of the kinase and having similar efficiency, but promote eIF2α phosphorylation by a mechanism in which acidic proteins are more efficient. Moreover, as it has been previously observed for the activation of GCN2 by viral RNA, our results support that auto-phosphorylation of the kinase *per se* is probably necessary, but not sufficient to provoke an effective phosphorylation of eIF2α [51].

Interestingly, all four strains assayed responded similarly to amino acid starvation by increasing eIF2α phosphorylation indicating that free P1/P2 proteins are not determinants of the modification of the initiation factor under these conditions, even though stalk proteins are better GCN2 activators in vitro than the deacylated tRNA, the natural effector [42]. These findings indicate that distinct mechanisms can regulate *S. cerevisiae* eIF2α phosphorylation in response to different stressors, and that the specific conditions determine the activator that participates in the process. This conclusion is illustrated in Figure 1, where the entry of cells in stationary phase, due to the consumption of glucose and nitrogen sources, induces a marked increase in eIF2α phosphorylation in wild-type cells, while only a modest effect is observed in D4567 cells probably because they only respond to an early amino acid deprivation, but not to the glucose starvation that seems to take place at higher optical density of the culture.

In this context, the size of the cytoplasmic pool of stalk proteins is obviously quite relevant. It is noteworthy that the absence of P1/P2 proteins significantly changes the rate of translation and the pattern of proteins synthesized in a yeast cell-free translation extract [11], and also a huge decrease of eIF2α phosphorylation, which is recovered by the addition of the stalk acidic proteins (Figure 4). There is, however, little information available regarding the changes in the cytoplasmic pool of P1/P2 proteins in response to growth conditions to support their proposed extra-ribosomal functions in wild-type cells. The overall and relative expression of different yeast acidic proteins changes with the carbon source [52]. Furthermore, over expression of protein P2β notably reduces the growth rate of cells, apparently by blocking the initiation of translation [53], and this result is compatible with an effect of the over-expressed protein on the eIF2α phosphorylation level. However, more detailed studies will be required to determine which specific stages of cell growth are blocked in order to confirm this possibility. In any case, the unexpected activity of the free stalk proteins must be taken into consideration when investigating the proposed regulatory functions of this essential ribosomal functional domain [2], [52]. In this respect, the increase of eIF2α phosphorylation in the wild-type strain at stationary phase, when the amount of P1/P2-deprived ribosomes, and consequently of P1/P2 free proteins, also increases [17], supports a coupling of the regulatory roles of the ribosomal stalk and the free stalk components.

## Materials and Methods

### Strains and Growth Conditions

The strains of *S. cerevisiae* used in this study are listed in Table 1. *S. cerevisiae* W303-1b is the wild-type parental strain of the previously described D45 [35], D67 [35] and D4567 [11] strains. *S. cerevisiae* J80 strain, lacking the *GCN2* gene, was provided by Dr. T. E. Dever (Laboratory of Gene Regulation and Development, National Institute of Child Health and Human Development, National Institutes of Health, Bethesda) and it has been described previously [54]. Unless otherwise indicated, yeast cells were grown at 30°C on rich YEP medium containing 2% glucose.

**Table 1:** Yeast strains.

Strain	Genotype	Missing protein	Reference
W303-1b	MATα, *leu2–3, trp1–1, ura3–1, ade2–1, his3–11,15, can 1–100*	None	Dr. Slonimski, Paris
D45	MATα, *leu2–3, trp1–1, ura3–1, ade2–1, his3–11,15, can 1–100, RPP2*α: *URA3*; *RPP2*β: *HIS3*	P2α, P2β	[35]
D67	MATα, *leu2–3, trp1–1, ura3–1, ade2–1, his3–11,15, can 1–100, RPP1*α: *LEU2*, *RPP1*β: *TRP1*	P1α, P1β	[35]
D4567	MATα, *leu2–3, trp1–1, ura3–1, ade2–1, his3–11,15, can 1–100, RPP1*α: *LEU2*, *RPP1*β: *TRP1,* *RPP2*α: *URA3*; *RPP2*β: *HIS3*	P1α, P1β, P2α, P2β	[11]
J80	MATa, *ura3–52, leu2–3, leu2–112, trp1–*Δ*63, gcn2*Δ, *sui2*Δ, *p[SUI2, LEU2]*	Gcn2	Dr. Dever, Bethesda

### Cell Transformation

*S. cerevisiae* transformations were performed by the Lithium acetate method [55].

### Stress Conditions

For glucose starvation, yeast cells growing at 30°C at mid-exponential phase (A_600_ = 0.3–0.4) in SD minimal medium supplemented with 2% glucose and the required amino acids were transferred to fresh medium containing either 2% or 0.05% glucose, and grown at 30°C. Control cells growing under non-starvation conditions (2% glucose) were harvested at A_600_ = 0.6, while starved cells (0.05% glucose) were collected after 4 h.

For osmotic stress, cells growing exponentially (A_600_ = 0.4) in 2% glucose SD medium were transferred to the same medium containing 0.5 M NaCl and grown for 1 h before harvesting.

For amino acid starvation, **c**ells growing exponentially (A_600_ = 0.6) at 30°C in minimal medium (0.67% YNB, 2% Glucose) supplemented with all the amino acids were collected and then transferred at the same concentration to same fresh medium in the presence or in the absence of amino acids. Cells were allowed to grow for 15 min at 30°C in a shaker before harvesting. Alternatively, cells growing at 30°C at mid-exponential phase (A_600_ = 0.6) in SD minimal medium supplemented with 2% glucose and the required amino acids were transferred to fresh medium containing 30 mM 3-amino-1,2,4-triazole (3-AT) for 1 h before harvesting.

### Plasmids

To obtain mouse wild-type GCN2 and other specific mutants (K618R, m2, ΔNt, ΔCt), HEK 293 cells were transfected with expression plasmids encoding these proteins. The pcMGCN2, pcMGCN2-K618R and pcMGCN2-m2 plasmids have been described previously [28]. To express the GCN2-ΔNt mutant, the pcMGCN2-ΔNt plasmid was constructed by deleting the nucleotides encoding the first 200 amino acids of the MGCN2 amino-terminus. For the GCN2-ΔCt mutant, the plasmid pcMGCN2-ΔCt was constructed by deleting the nucleotides encoding the last 156 amino acids of the MGCN2 carboxyl end. The corresponding coding sequences were cloned pCDNA3.1 Myc-His, encoding the Myc epitope and a 6xHis track at the 3′ end of the cDNA.

Plasmid p585, which encodes a wild-type GCN2 gene [56], was a generous gift from Dr C. R. Vazquez de Aldana. Other plasmids used to transform yeast cells were pFL36 [57], pFL36 P2α [58] and pFL36 P2β [37].

### Cell Fractionation

Cell pellets were resuspended in lysis buffer (20 mM Tris-HCl pH 7.6, 150 mM NaCl, 10% glycerol, 1 mM EDTA, 1 mM DTT, 1% (v/v) Triton-X100, 25 µg ml^−1^ DNase, protease and phosphatase inhibitor cocktail [Complete® and Phostop®, Roche]) and broken up with acid-washed glass beads (Sigma) in a FastPrep®-24 (MP Biomedicals) over three 32 second cycles at a power setting of 6. The lysates were clarified by centrifugation at 12,000×g at 4°C for 15 min and stored at −75°C. The protein concentration was determined using a Bio-Rad protein assay according to the manufacturer’s instructions. In some experiments cells were lysed using a different protein extraction method, as described previously [59].

Ribosomes were prepared by centrifugation of the cell lysates at 100,000×g for 2 h at 4°C. The pelleted ribosomes were washed by centrifugation through a discontinuous 20% and 40% sucrose gradient in 20 mM Tris-HCl [pH 7.4], 100 mM MgCl_2_, 0.5 M NH_4_Cl and 5 mM 2-mercaptoethanol, and resuspended in 10 mM Tris-HCl [pH 7.4], 20 mM KCl, 12.5 mM MgCl_2_, and 5 mM 2-mercaptoethanol, supplemented with a protease inhibitor cocktail. The acidic P1/P2 proteins (SP_0.3_ fraction) were extracted from the ribosomes by treatment with 0.3 M NH_4_Cl/50% ethanol [26]. The extracted fraction was dialysed against 10 mM Hepes pH 7.4, 200 mM K(OAc), 1 mM Mg(OAc)_2_ and 0.5 mM PMSF, and concentrated by filtration through centricon SR3 membranes (Amicon) [11].

Yeast cell-free translation extract was obtained as previously described [11].

### Protein Purification

Purification of Myc and 6xHis-tagged HRI, PKR, GCN2 full-length and GCN2 mutants (GCN2-K618R, GCN2-m2, GCN2-ΔNt, GCN2-ΔCt) was done as previously described [28].

Recombinant proteins P1α and P2β were purified from *E. coli* BL21(DE3)pLys previously transformed with a pT7-7 vector carrying the corresponding yeast genes under the control of the T7 promoter, as described previously [39].

### Protein Analysis

Aliquots of cell extracts (50 µg of protein) were separated by 5–18% gradients sodium dodecyl sulphate-polyacrylamide gel electrophoresis (SDS-PAGE) and transferred onto polyvinylidene fluoride (PVDF) membranes (Immobilon-P; Millipore). The membranes were probed with the following antibodies: rabbit anti-eIF2α (kindly provided by Dr T.E. Dever, Laboratory of Gene Regulation and Development, National Institute of Child Health and Human Development, National Institutes of Health, Bethesda); rabbit anti-eIF2α-P (Cell Signaling); rabbit anti-GCN2 phospho-Thr898 (Cell Signaling, Abcam); rabbit anti-Gcn2 (kindly provided by Dr A.G. Hinnebusch, Laboratory of Gene Regulation and Development, National Institute of Child Health and Human Development, National Institutes of Health, Bethesda); and a monoclonal 3BH5, specific for yeast stalk proteins [60]. Horseradish peroxidase-conjugated rabbit or mouse secondary antibodies were used (Promega) and after extensive washing, the immunoreactive bands were detected by enhanced chemiluminescence (ECL, GE Healthcare).

### In Vitro Kinase Activity

Affinity-purified GCN2 (wild-type or mutants), PKR and HRI [28], [61], were assayed for their ability to phosphorylate purified rabbit reticulocyte eIF2, in the presence or absence of tRNA (Sigma), polysomal RNA [62], Sindbis virus RNA (SV RNA) and purified acidic stalk P proteins. Briefly, in a total volume of 20 µl, affinity-purified kinases were incubated, without or with RNAs or polysomal RNA and/or purified acidic stalk P proteins at various concentrations, for 30 min at 30°C in kinase buffer (20 mM Tris–HCl pH 7.6, 2.5 mM MgCl_2_, 2.5 mM Mg(OAc)_2_, 0.25 mg/ml BSA, 50 mM ATP including 0.5 µg of purified rabbit reticulocyte eIF2). Kinase reactions were stopped by the addition of SDS-PAGE loading buffer and the proteins were analysed in Western blots using specific antibodies [28].

## Supporting Information

Figure S1 Stimulation of eIF2α phosphorylation in conditions of osmotic stress and glucose starvation is dependent on the presence of GCN2. The J80 strain of *S. cerevisiae*, which lacks the *GCN2* gene, and the J80 strain transformed with the plasmid p585, which contains a copy of the *GCN2* gene (J80+p585), were grown under conditions of osmotic stress (in the presence of 0.5 M NaCl: A) or low glucose (0.05%: B). Extracts from the stressed cells and the corresponding unstressed control cells were resolved, and the indicated proteins were analyzed by Western blot with the corresponding specific antibodies as described in previous figures. Similar results were obtained from duplicate experiments.
(TIF)

Figure S2 Response of *S. cerevisiae* stalk mutants to the amino acid deprivation induced by 3-amino-1,2,4-triazole (3-AT) treatment. Yeast D45, D67 and D4567 and the parental W303-1b (WT) strains were grown in the presence (+) or absence (–) of 30 mM 3-AT for 1 h as described in the Materials and methods section. After the treatment, cells were collected, the total cell extracts were resolved by SDS-PAGE and the amount of phosphorylated and total eIF2α was analyzed as described in previous figures. The values under Western blot panels represent the intensities of phosphorylated eIF2α in each lane normalized respect to the corresponding total eIF2α; for comparison, the values obtained for the untreated (–) WT cells was set as 1. Similar results were obtained from duplicate experiments.
(TIF)
