# Supplementary figures and images for: Phosphorylation of Initiation Factor eIF2 in Response to Stress Conditions Is Mediated by Acidic Ribosomal P1/P2 Proteins in Saccharomyces cerevisiae
Source: PLoS One. 2013 Dec 31;8(12):e84219. doi: 10.1371/journal.pone.0084219 (PMC3877244; doi:10.1371/journal.pone.0084219)

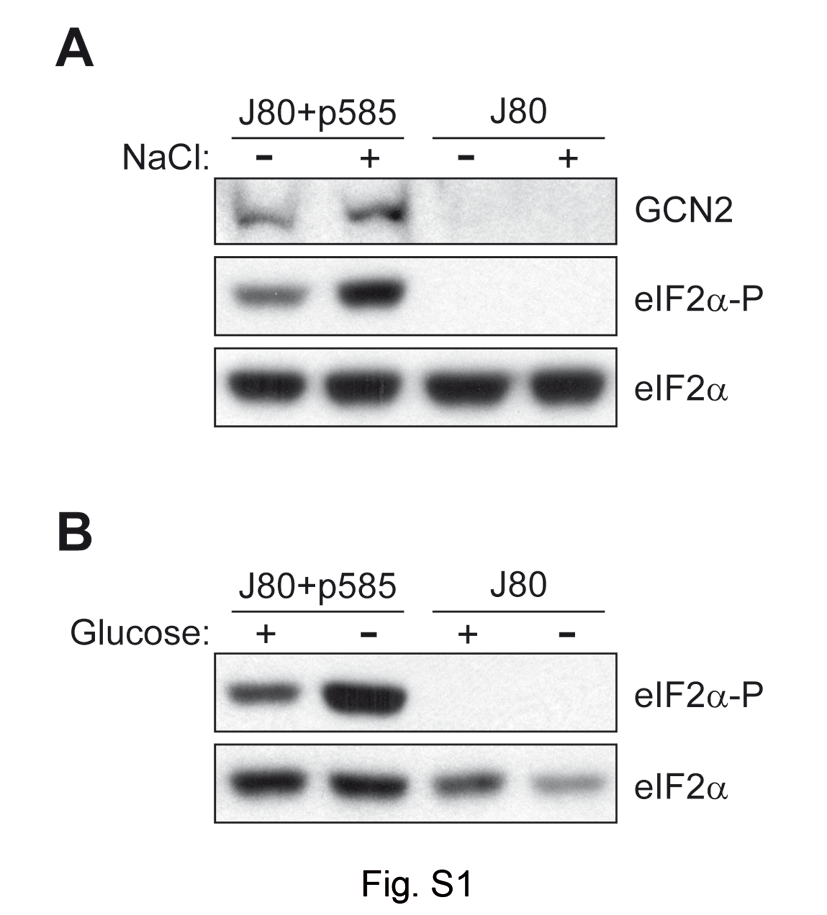

Supplement: Figure S1 — Stimulation of eIF2α phosphorylation in conditions of osmotic stress and glucose starvation is dependent on the presence of GCN2. The J80 strain of S. cerevisiae, which lacks the GCN2 gene, and the J80 strain transformed with the plasmid p585, which contains a copy of the GCN2 gene (J80+p585), were grown under conditions of osmotic stress (in the presence of 0.5 M NaCl: A) or low glucose (0.05%: B). Extracts from the stressed cells and the corresponding unstressed control cells were resolved, and the indicated proteins were analyzed by Western blot with the corresponding specific antibodies as described in previous figures. Similar results were obtained from duplicate experiments. (TIF) [file pone.0084219.s001.tif]

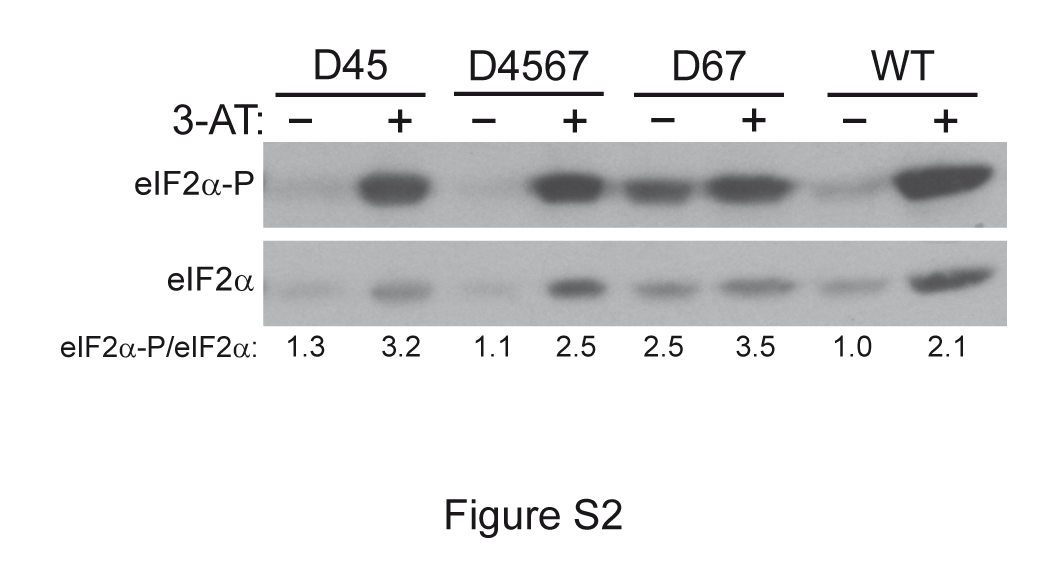

Supplement: Figure S2 — Response of S. cerevisiae stalk mutants to the amino acid deprivation induced by 3-amino-1,2,4-triazole (3-AT) treatment. Yeast D45, D67 and D4567 and the parental W303-1b (WT) strains were grown in the presence (+) or absence (–) of 30 mM 3-AT for 1 h as described in the Materials and methods section. After the treatment, cells were collected, the total cell extracts were resolved by SDS-PAGE and the amount of phosphorylated and total eIF2α was analyzed as described in previous figures. The values under Western blot panels represent the intensities of phosphorylated eIF2α in each lane normalized respect to the corresponding total eIF2α; for comparison, the values obtained for the untreated (–) WT cells was set as 1. Similar results were obtained from duplicate experiments. (TIF) [file pone.0084219.s002.tif]
